# Patch Testing Is Useful in Patients With Lamotrigine‐Induced Cutaneous Adverse Drug Reactions

**DOI:** 10.1111/cod.70219

**Published:** 2026-07-20

**Authors:** E. Bertazzo, N. Laviec, C. Toussaint, P. Sanchez‐Pena, B. Milpied

**Affiliations:** ^1^ Department of Dermatology Poitiers University Hospital Poitiers France; ^2^ Department of Pharmacovigilance Bordeaux University Hospital Bordeaux France; ^3^ Department of Dermato‐Allergology Bordeaux University Hospital Bordeaux France

**Keywords:** cutaneous hypersensitivity drug reactions, lamotrigine, patch test

Lamotrigine (LT) is widely used for epilepsy and bipolar disorders but may induce severe cutaneous adverse drug reactions (SCARs), including DRESS and epidermal necrolysis [1]. In patients receiving multiple drugs, identifying the culprit medication remains challenging, and skin eruptions occurring during LT treatment do not necessarily indicate true hypersensitivity [2]. Patch testing can help document delayed‐type drug allergy [3], but available data for LT are limited [4], and optimal test concentrations and vehicles remain poorly standardized [5].

## 1 Materials and Methods

We retrospectively reviewed patients patch tested for suspected LT‐induced cutaneous adverse drug reactions between 2011 and 2024. Patch tests were performed according to European Society of Contact Dermatitis guidelines, with readings on Days 2, 3 and 7 if initially negative. LT was tested as active ingredient 10% petrolatum (LT 10% pet.; Chemotechnique Diagnostics) and as the patient's own commercial preparation diluted to 30% in petrolatum (commercial 30% pet.), water (commercial 30% aq.) or saline (commercial 30% saline).

Thirty patients were included. Most were women (22/30) and LT was mainly prescribed for mood stabilization (24/30). The main clinical phenotypes were DRESS (11/30), maculopapular exanthema (10/30) and Stevens–Johnson syndrome (5/30).

Not all preparations were tested in every patient, reflecting the retrospective design and changes in available preparations over time. Twenty‐one were tested with LT CD 10% pet., 29 with the commercial 30% pet., 25 with the commercial 30% aq. and 2 with the commercial 30% saline preparation. Tests that were not performed, as well as those with missing data in the medical records, were classified as not performed (NP). The day of positivity was unavailable for most patients; however, one delayed positive reaction at D7 was documented (Table 1).

**TABLE 1:** Characteristics of patients with suspected lamotrigine‐induced CADRs.

Patient	Sex	Age (years)	Clinical phenotype	PT LT 10% pet. (CD)	PT com 30% pet.	PT com 30% aq.	PT com 30% sal.
1	F	21	SJS	NP	N	P	NP
2	F	40	EMP	NP	N	P	NP
3	F	33	DRESS	NP	N	N	NP
4	F	47	EMP	NP	NP	N	NP
5	F	33	DRESS	NP	P	P	NP
6	F	53	DRESS	NP	N	N	NP
7	F	46	DRESS	NP	N	N	NP
8	F	49	UR	N	N	N	NP
9	M	30	DRESS	P	P	P	NP
10	F	39	ND	NP	N	N	NP
11	F	72	DRESS	N	P	N	NP
12	M	76	DRESS	N	N	NP	NP
13	M	46	EMP	N	N	N	NP
14	M	47	DRESS	N	N	N	NP
15	F	18	EMP	P	P	N	NP
16	F	18	EMP	N	N	N	NP
17	F	35	EM	N	N	N	NP
18	F	36	SJS	N	N	N	NP
19	M	70	SJS	N	N	P	NP
20	F	34	DRESS	N	N	N	NP
21	F	44	EMP	NP	N	N	NP
22	F	37	UR	N	N	N	NP
23	M	87	EMP	N	N	N	NP
24	M	53	EMP	P	P	NP	NP
25	M	62	EMP	P	P	NP	NP
26	F	71	DRESS	N	N	NP	NP
27	F	53	SJS	N	N	NP	NP
28	F	34	SJS	N	N	N	NP
29	F	66	DRESS	N	N	N	N
30	F	83	EMP	N	P	P	N

Abbreviations: Com 30% aq., commercial preparation diluted to 30% in water; Com 30% pet., commercial preparation diluted to 30% in petrolatum; Com 30% sal., commercial preparation diluted to 30% in normal saline; DRESS, drug reaction with eosinophilia and systemic symptoms; EM, erythema multiforme; EMP, maculopapular exanthem; F, female; M, male; LT 10% pet. (CD), lamotrigine 10% petrolatum (Chemotechnique Diagnostics); N, negative; NP, not performed; P, positive; PT, patch test; SJS, Stevens–Johnson syndrome; UR, urticaria.

Among the 30 patients, 10 (33%) had at least one positive patch test result. Positivity rates were 70% (7/10) for commercial 30% pet., 60% (6/10) for commercial 30% aq. and 40% (4/10) for LT 10% pet. Commercial 30% saline was negative in both tested patients. Only one patient reacted to all three preparations, whereas five reacted to two preparations. No clear association was observed between clinical phenotype and patch test positivity (Table 2).

**TABLE 2:** Characteristics of patients with suspected lamotrigine‐induced CADRs and positive patch test results.

Patient	Sex	Age (years)	Clinical phenotype	Onset after introduction, increasing dose (days)	Indication	Dose (mg)		Dose increase	PT LT 10% pet. (CD)	PT com 30% pet.	PT com 30% aq.	PT com 30% sal.
1	F	21	SJS	15, 7	E	50		Yes	NP	N	P	NP
2	F	40	EMP	7, 2	T	50		Yes	NP	N	P	NP
5	F	33	DRESS	50, 26	T	100		Yes	NP	P	P	NP
9	M	30	DRESS	30, ND	T	100		No	P	P	P	NP
11	F	72	DRESS	ND	T	ND		ND	NP	P	N	NP
15	F	18	EMP	15, ND	T	ND		Yes	P	P	N	NP
19	M	70	SJS	21, ND	E	ND		ND	N	N	P	NP
24	M	53	EMP	14, ND	T	100		Yes	P	P	N	NP
25	M	62	EMP	60, ND	E	ND		Yes	P	P	N	NP
30	F	83	EMP	35, ND	T	50		Yes	N	P	P	N

Abbreviations: Com 30% aq., commercial preparation diluted to 30% in water; Com 30% pet., commercial preparation diluted to 30% in petrolatum; Com 30% sal., commercial preparation diluted to 30% in normal saline; DRESS, drug reaction with eosinophilia and systemic symptoms; EMP, maculopapular exanthem; F, female; M, male; LT 10% pet. (CD), lamotrigine 10% petrolatum (Chemotechnique Diagnostics); N, negative; ND, not determined; NP, not performed; P, positive; PT, patch test; SJS, Stevens–Johnson syndrome.

## 2 Discussion

Patch testing is increasingly used to support the diagnosis of delayed‐type hypersensitivity reactions to antiepileptic drugs, although sensitivity varies according to the drug, clinical phenotype and testing methodology [6, 7]. Data specifically addressing LT remain limited [4, 8, 9, 10].

In our cohort, one‐third of patients had at least one positive patch test reaction, consistent with previous reports [11]. Commercial preparations yielded more positive results than LT 10% pet., and aqueous and petrolatum preparations appeared complementary, each identifying additional positive cases. These findings support the importance of vehicle selection in LT patch testing and may partly explain discrepancies across published studies. No positive reactions were observed with commercial 30% saline, although only two patients were tested. No clear association between clinical phenotype and patch test positivity was identified, likely because of the limited sample size.

The retrospective single‐centre design and non‐systematic use of all preparations limit direct comparison between vehicles. Nevertheless, to our knowledge, this is the first study specifically evaluating LT patch testing in a dedicated cohort.

In conclusion, LT patch testing may help identify LT as the culprit drug in suspected delayed cutaneous adverse reactions. Combining commercial LT preparations in petrolatum and water may improve sensitivity, while LT active ingredient 10% in petrolatum helps confirm drug‐specific sensitization. Larger prospective multicentre studies are needed to standardize LT patch testing procedures.

## Author Contributions

**B. Milpied:** writing – review and editing, project administration, validation, conceptualization, supervision. **N. Laviec:** investigation, resources. **C. Toussaint:** validation. **E. Bertazzo:** writing – original draft, investigation. **P. Sanchez‐Pena:** methodology, validation.

## Conflicts of Interest

The authors declare no conflicts of interest.

## Data Availability

The data that support the findings of this study are available from the corresponding author upon reasonable request.
